# Characterization of the human TARDBP gene promoter

**DOI:** 10.1038/s41598-021-89973-z

**Published:** 2021-05-17

**Authors:** Marco Baralle, Maurizio Romano

**Affiliations:** 1 International Centre for Genetic Engineering and Biotechnology (ICGEB), Area Science Park, Padriciano, Trieste, Italy; 2 Department of Life Sciences, University of Trieste, Via A. Valerio 28, 34127 Trieste, Italy

**Keywords:** Amyotrophic lateral sclerosis, Dementia, Mechanisms of disease, Gene regulation, Gene expression

## Abstract

The expression of TDP-43, the main component of neuronal intracellular inclusions across a broad spectrum of ALS and FTD disorders, is developmentally regulated and studies in vivo have shown that TDP-43 overexpression can be toxic, even before observation of pathological aggregates. Starting from these observations, the regulation of its expression at transcriptional level might represent a further key element for the pathogenesis of neurodegenerative diseases. Therefore, we have characterized the human TARDBP promoter, in order to study the transcriptional mechanisms of expression. Mapping of cis-acting elements by luciferase assays in different cell outlined that the activity of the promoter seems to be higher in SH-SY5Y, Neuro2A, and HeLa than in HEK293. In addition, we tested effects of two SNPs found in the promoter region of ALS patients and observed no significant effect on transcription levels in all tested cell lines. Lastly, while TDP-43 overexpression did not affect significantly the activity of its promoter (suggesting that TDP-43 does not influence its own transcription), the presence of the 5′UTR sequence and of intron-1 splicing seem to impact positively on TDP-43 expression without affecting transcript stability. In conclusion, we have identified the region spanning nucleotides 451–230 upstream from the transcription start site as the minimal region with a significant transcription activity. These results lay an important foundation for exploring the regulation of the TARDBP gene transcription by exogenous and endogenous stimuli and the implication of transcriptional mechanisms in the pathogenesis of TDP-43 proteinopathies.

## Introduction

TAR DNA-Binding Protein 43 (TDP-43) is an ubiquitously expressed and highly conserved nuclear protein encoded by the human TARDBP gene, and it is involved in several cellular mechanisms, including transcription, pre-mRNA processing, splicing and translation^1,2^. After initial implication in the pathogenesis of monosymptomatic forms of cystic fibrosis^3–7^, it has been discovered a direct association between TDP-43 abnormalities and neurodegenerative diseases, and in particular with Amyotrophic Lateral Sclerosis (ALS) and Frontotemporal Degeneration (FTD)^8,9^ was discovered. Over the last years, TDP-43 immunoreactive inclusions have also been reported in 70% of patients with hippocampal sclerosis, 30% of patients with Alzheimer's disease, 33% of patients with Pick's disease and in a subset of patients with Lewy-body related disease^10–17^. Whereas many efforts have been expended in characterizing TDP-43 pathophysiological functions, a contentious debate ensued and is still ongoing over the possible mechanisms whereby TDP-43 triggers neurodegeneration. It has been proposed both that cytoplasmic aggregates might be neurotoxic ("gain of function" hypothesis) and that the sequestration of the factors in aggregates might induce a functional deficiency resulting in alteration of the TDP-43-regulated processes ("loss of function" hypothesis)^18–22^. Of course, the two hypotheses are not necessarily mutually exclusive, and studies with animal models have shown that TDP-43 overexpression can be neurotoxic, even without presence of inclusions in worms^23^, flies^24–26^, mice^27–33^, and monkeys^34^. Importantly, mice expressing the mutant A315T TDP-43 mice show a dose-dependent degeneration of cortical and spinal motor neurons, without evidence of cytoplasmic TDP-43 inclusions^32^. These studies suggest that even an increase in TDP-43 can contribute to neurodegeneration without occurrence of aggregation. In addition, elevated levels of TDP-43 has been found in the cerebrospinal fluid^35–37^ as well as in plasma of ALS^38–40^, FTD and Alzheimer^41,42^ patients, and in FTD patients carrying C9orf72 expansion or GRN mutations, plasma phosphorylated TDP-43 levels have been found to be higher than in healthy controls^43^. On the other hand, it is now well established TDP-43 levels are tightly controlled: in fact, it has been demonstrated that TDP-43 can regulate its own expression through a negative feedback loop^44–51^. Altogether these observations suggest that regulation of TDP-43 expression at transcriptional level might also be implicated in the pathogenesis of neurodegenerative diseases.

The study of the TARDBP gene promoter is therefore useful to shed further light on the pathogenesis of TDP-43 proteinopathies. Interestingly, two SNPs were found within the promoter sequence of TARDBP gene in ALS patients^52^. However, no functional studies have been so far carried out.

In this work, we have characterized the cis-acting elements important for TARDBP gene regulation and evaluated its cell-type specificity. Subsequently, we have characterized the possible impact of some cis-acting elements (SNPs and 5′UTR), trans-acting factors (TDP-43 itself) and events (splicing) on the transcriptional activity of TARDBP promoter.

## Results

### Bioinformatic analysis of the *TARDBP* promoter

**Figure 1:**
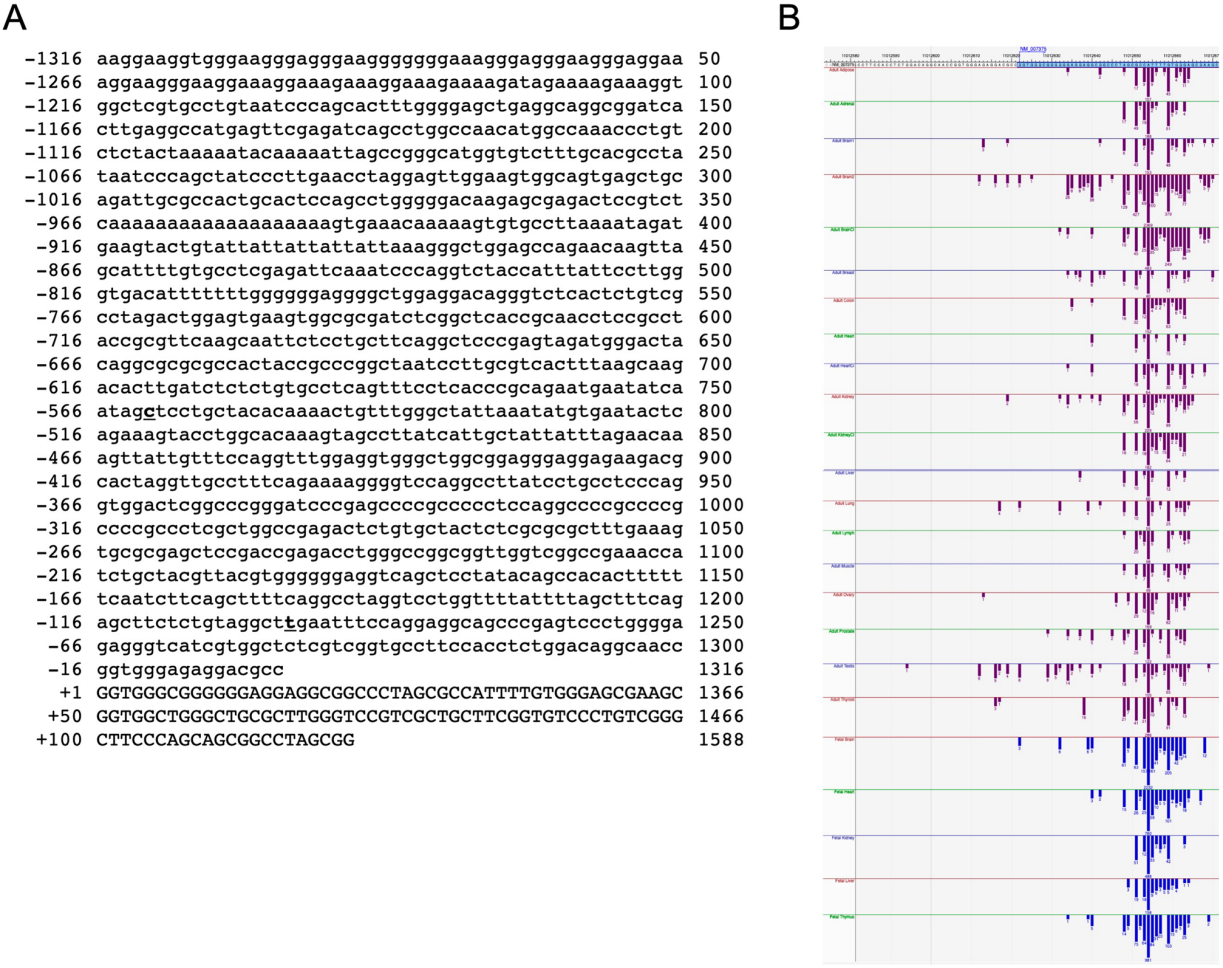
Sequence of the human TARDBP promoter and map of the TARDBP TSSs in adult and fetal tissues. (**A**) Proximal sequences (− 1316/ + 122) of the human TARDBP promoter are shown. Transcript reference: NM_007375.4. The promoter sequences are numbered (left side) relative to the transcription start site (TSS, + 1). (**B**) Graphical overview of the multiple TARDBP TSSs mapped in adult and fetal tissues presented in the DataBase of Transcriptional Start Sites (DBTSS). The top turquoise (5′-region) box correspond to the RefSeq NM_007375. Red boxes represent DBTSS clones of Adult tissues, while blue boxes represent DBTSS clones of Fetal tissues.

The characterization of *TARDBP* gene sequence (Ensembl gene ID: ENSG00000120948) was initially performed by a bioinformatic analysis using the UCSC Genome Browser (GRCh37/hg19). The *TARDBP* gene, mapped to chromosome 1p36.22 region, spans 12870 bp from position 11,072,679 to 11,085,548 on the forward DNA strand. Figure 1 shows the TARDBP promoter sequence (starting from the 1316 nucleotide upstream from the TSS of NM_007375.3 TDP-43 transcript). Inspection of UCSC TARDBP transcripts matched with those retrieved from the Database of Transcriptional Start Site (DBTSS, http://dbtss.hgc.jp^53,54^), most of transcription start sites (TSS) mapped at the corresponding positions indicated in the Reference Sequence NM_007375 (Fig. 1A,B). Apparently, there are no tissue-specific TSSs, since only one mRNA species seems to be predominant in Adult and Fetal Tissues, as well as in different tissues (Fig. 1B).

Subsequently, by querying the Eukaryotic Promoter Database^55^, different putative TATA-box motifs ([p-value = 0.01]: − 930, − 865, − 606, − 571, − 513, − 309, upstream of TSS shown in Fig. 1A), CG-box sequences (CCAAT-box [p-value = 0.01]: − 744, -677, − 572, − 61, upstream of TSS shown in Fig. 1A) were identified, but deeper inspection revealed that the retrieved sequences were not consistent either with the canonical human promoter motifs or with the main mapped TSS (DBTSS, http://dbtss.hgc.jp, Fig. 1). In addition, a further analysis performed with another promoter search tool (GPMiner^56^) failed to detect the main core promoter elements. These observations suggest that the expression of the human TARDBP gene is driven by a TATA-less promoter.

### Conservation of TARDBP promoter sequence throughout evolution

**Figure 2:**
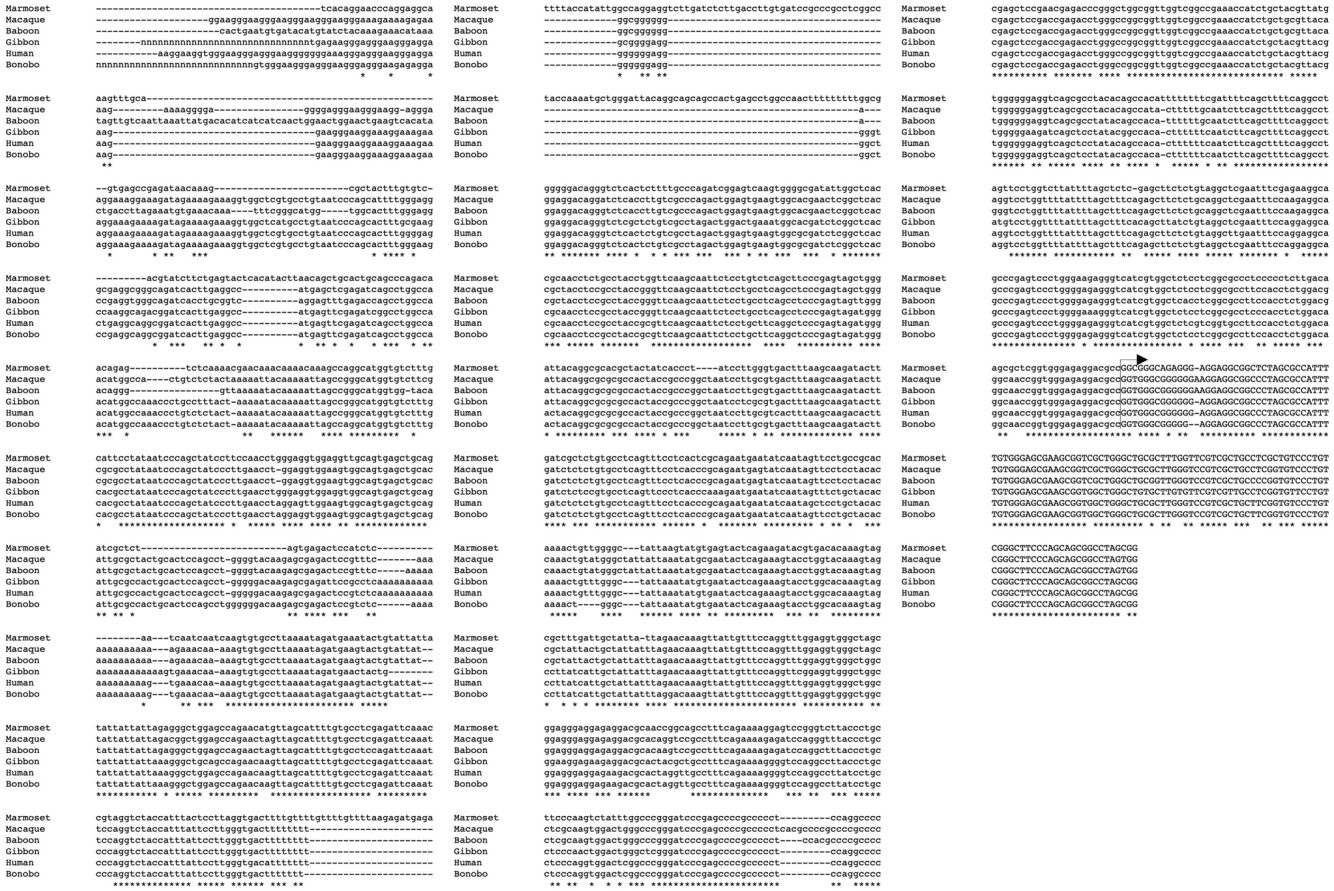
Genomic alignment of putative promoter regions from Homo sapiens, Pan troglodytes and Callithrix jacchus TARDBP genes. The alignment of the 1316nt—sequence upstream and 78 bp downstream of the transcription starting site of Human (Homo sapiens, ENSG00000120948, NM_007375.3) was carried out versus: Marmoset (Callithrix jacchus, ENSCJAG00000002381), Macaque (Macaca mulatta, ENSMMUG00000007456), Olive baboon (Papio anubis, ENSPANG00000017459), Gibbon (Nomascus leucogenys, ENSNLEG00000009512) and Bonobo (Pan paniscus, ENSPPAG00000039086) TARDBP transcripts, by using the MUSCLE alignment program (http://www.ebi.ac.uk/Tools/muscle/index.html).

**Figure 3:**
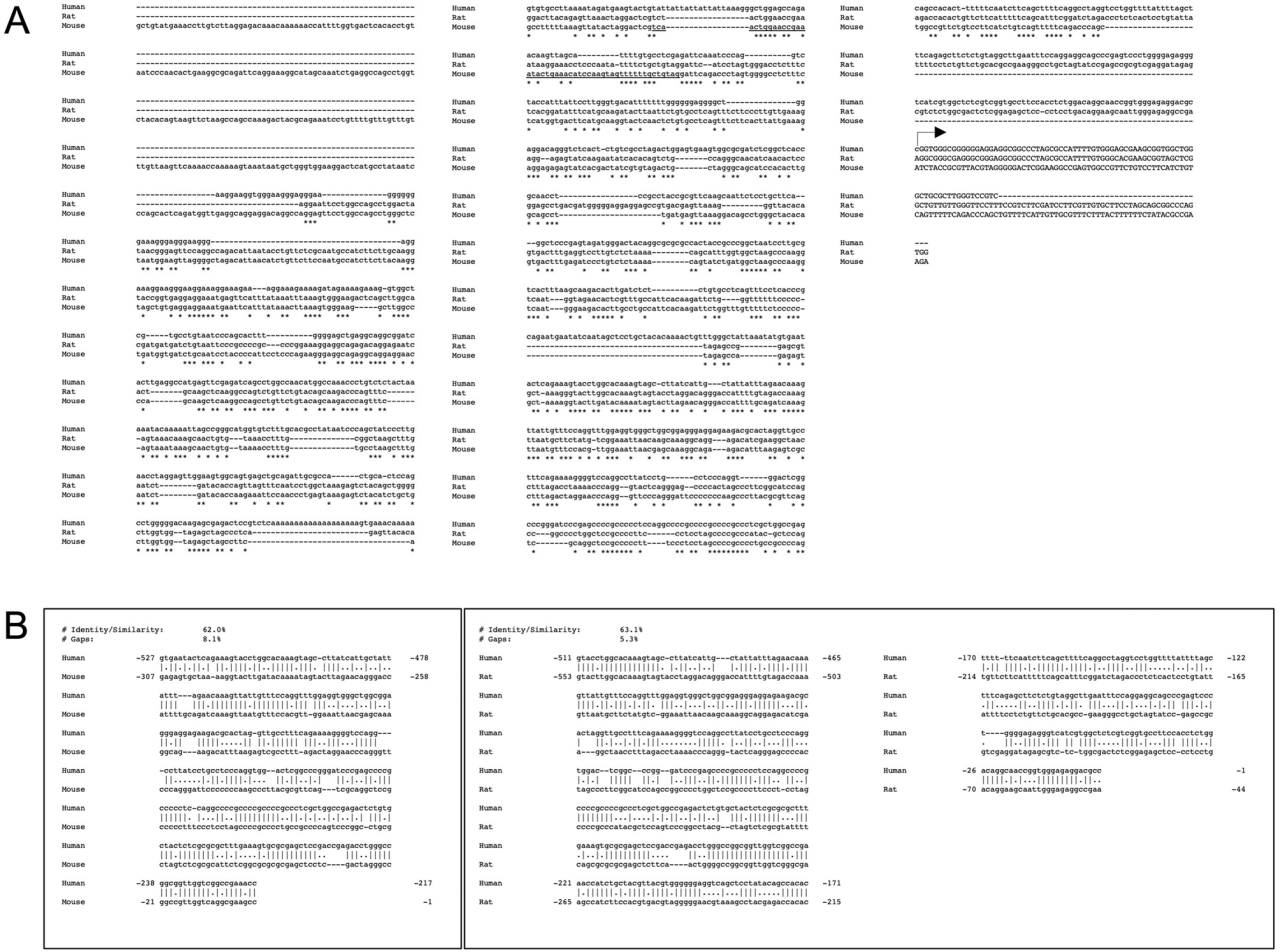
Global and local alignment of putative promoter regions from Homo sapiens, Mus musculus, Rattus norvegicus and Drosophila melanogaster TARDBP ortholog genes. (**A**) The alignment of the 1316nt—sequence upstream of the transcription starting site of Human (RefSeq: NM_007375.3), Mouse (ENSMUST00000084125) and Rat (ENSRNOT00000049822) TARDBP transcripts was generated with MUSCLE alignment software (http://www.ebi.ac.uk/Tools/muscle/index.html). (**B**) Pairwise Sequence Alignment was used to identify regions of identity/similarity between the promoter sequences of the Human/Mouse and Human/Rat TARDBP orthologs. The local similarities between the sequences were identified by using the EMBOSS Matcher Local alignment tool (https://www.ebi.ac.uk/Tools/psa/).

In order to study the evolutionary conservation of TARDBP promoter, the 1316 nucleotides upstream of the TSS of TARDBP transcripts from human and other species were retrieved using GenBank and Ensembl Genome Browser (http://www.ensembl.org/). Alignments were performed using MUSCLE software^57^. Among primates, we compared the genomic sequences of the putative TARDBP promoter region from Hominoidea (*Homo sapiens*—Human, *Pan paniscus*—Bonobo, and *Nomascus leucogenys*—Gibbon), from of New World monkey (*Callithrix jacchus*—Common marmoset) and from Old World monkey (*Macaca mulatta*—Rhesus macaque; *Papio anubis*—Olive baboon). In general, this comparison highlighted that the relative degree of similarity among all three species increases significantly with respect to the proximity of the TSS. Then, in the region spanning from nucleotide -1316 to -1000 upstream from TSS, the hominoid sequences seem to be closer to each other than with that of New and Old World monkeys. On the other hand, in the region spanning the 1000 nucleotides upstream from TSS, the sequences from all primates show a high degree of identity (Fig. 2). On the other hand, the alignment of the human, mouse and rat putative TARDBP promoter sequences shows a low degree of global similarity (Fig. 3A). Nevertheless, the region of the rodent gene promoters spanning approximately 500nt upstream from the TSS shows a higher level of similarity, suggesting that proximal region of the putative TARDBP promoter region might encompass regulatory elements conserved across primates and rodents. In fact, pairwise sequence alignment between the 1316nt-sequence upstream of TSS of the human/mouse and human/rat TARDBP orthologous genes outlined a 62%/63% of local similarity/identity (with 8% and 5% gaps, respectively) between the sequences of the two species, in 300/500nt-region upstream of the respective TSSs (Fig. 3B). In consideration of the observations pinpointing how age regulated variations in TDP-43 expression seem to be evolutionary conserved at least in mouse and fly^58,59^, we tried to perform pairwise sequence alignment between the 1316nt-sequence upstream of TSS of the mouse and fruit fly TARDBP ortholog genes. The local alignment of two the sequences returned a low % of global identity/similarity (% Identity/Similarity: 41.4%, % Gaps: 46.8%), without outlining regions evolutionary conserved between these two species (data not shown).

### Characterization of cis-regulatory sequences of TARDBP promoter

**Figure 4:**
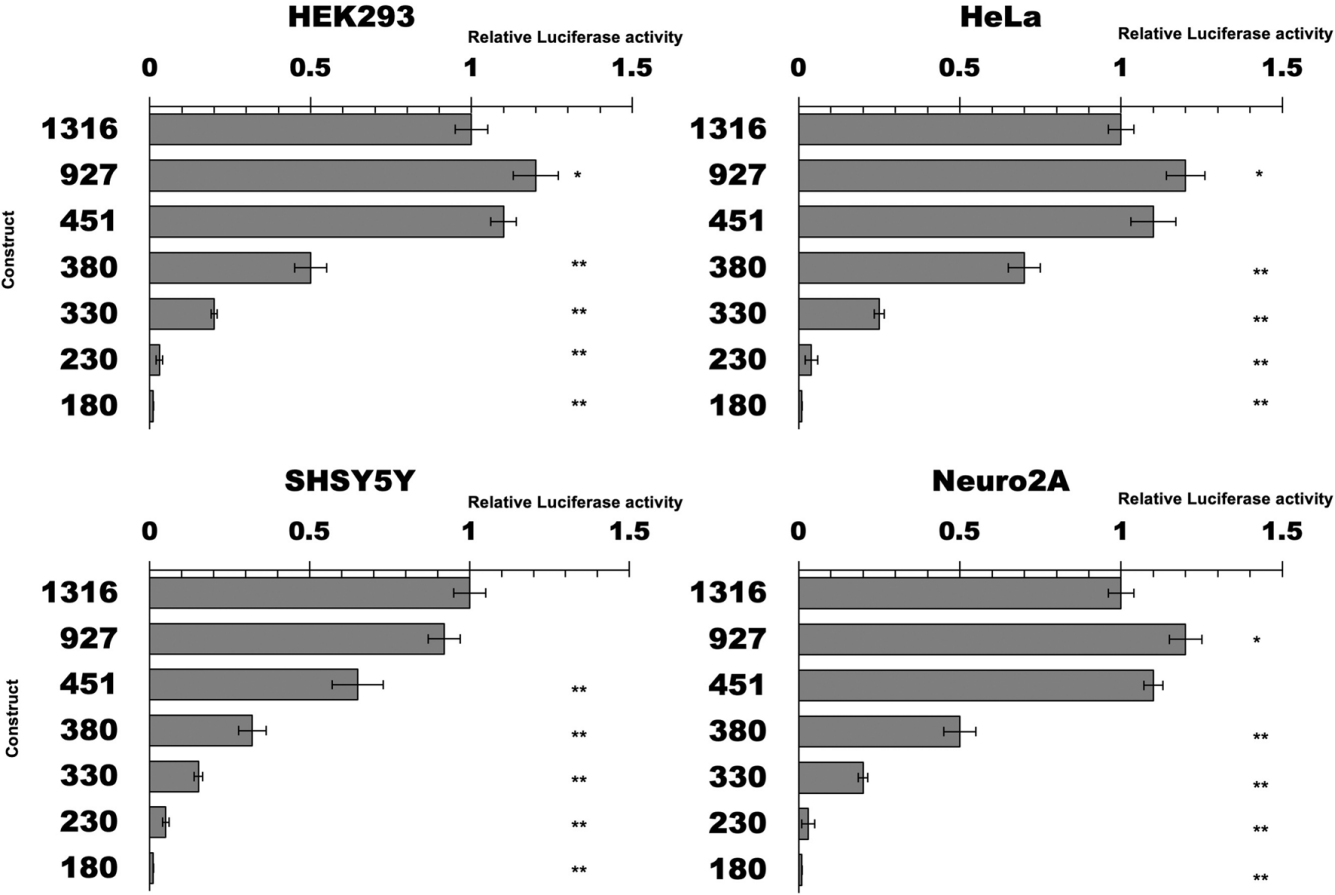
Deletion analysis of the human TARDBP promoter. Firefly luciferase activity of TARDBP promoter deletants in different cell lines (HEK293, HeLa, SH-SY5Y and Neuro2A) was measured in the cell lysate and values were normalized against Renilla. Activity of deletants is expressed as-fold against the cells transfected with the 1316 construct (= 1). Bars indicate the mean value of three independent assays. Error bars indicate standard deviation: *p < 0.05, and **p < 0.01 (One way ANOVA with Tukey test).

In order to identify the minimal functional sequence responsible for the transcriptional activity of the promoter of the human TARDBP gene, a luciferase assay was set up with deletion-fragments of the putative TARDBP promoter constructs cloned into the pGL4 luciferase reporter vector. Different segments of the putative regulatory region of the promoter were amplified through a PCR reaction, and eight constructs differing in length were produced: the sequences encompassed between 27 nucleotides downstream from the TSS (+ 1nt, RefSeq NM_007375.3) to 1316, 927, 451, 380, 320, 280, 230 and 180 nucleotides upstream from the TSS (Fig. 4).

The promoter fragments were subcloned into the pGL4.11 vector, transfected in cell lines of different tissue origin (HEK293, HeLa, Neuro2A and SH-SY5Y) and the luciferase activity of each construct was normalized versus the 1316 construct (i.e., 1316 = 1).

Intracellular analysis showed that most of the promoter activity was retained in the fragments spanning from 1316 to 451 nucleotides upstream from the TSS within all tested cell lines (Fig. 4). Further deletions resulted in dramatic reduction of activity (380 and 330 constructs), with almost complete loss of activity when the promoter was shortened to 230 and 180 nucleotides upstream from the TSS, in all tested cell lines. These results show that the minimal promoter region encompasses the 451 nucleotides upstream from the TSS of RefSeq NM_007375.

**Figure 5:**
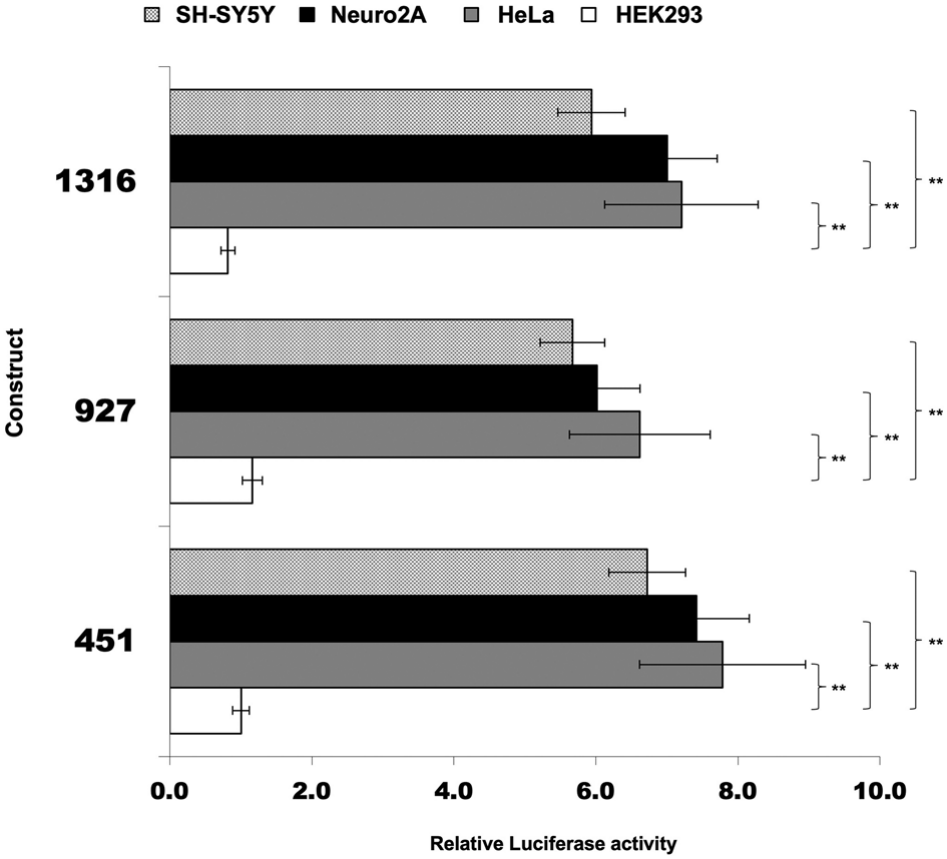
Comparison of the human TARDBP promoter activity in different cell lines. Firefly luciferase activity of 1316, 927 and 451 constructs was tested (normalized against Renilla) in different cell lines (HEK293, HeLa, SH-SY5Y and Neuro2A). Activity is expressed as-fold against transfections in HEK293 cells (= 1). Data are mean (± SD) of three independent assays: *p < 0.05, and **p < 0.01 (One way ANOVA with Tukey test).

We also tried to detect any kind of tissue-specific promoter activity. To this aim, we compared the activity of the 1316, 927 and 451 promoter fragments among SH-SY5Y, Neuro2A, HeLa, and HEK293 cell lines, considering the HEK293 cells as the unitary reference. Indeed, the constructs showed approximately 6–8 × higher activity both in two neuronal cell lines (SH-SY5Y, Neuro2A) and in HeLa cervical carcinoma cell line than in HEK293 embryonic kidney cells (Fig. 5).

### Transcriptional effects of SNPs found within the TARDBP promoter of ALS patients

The analysis of pathogenic mutations found in the *TARDBP* gene sequence of 46 Australian patients of European descent affected by sporadic Amyotrophic Lateral Sclerosis (sALS) has revealed two promoter variants (c.1-562t>c and c.1-100t>c) with a different frequency in patients than in controls (115 neurologically normal people or HapMap European and Sub-Saharan African cohorts)^52^. The c.1-562t>c single nucleotide substitution (rs9430335; NG_008734.1:g.4439CT; NM_007375.3:c.-696C>T) was found in homozygosity (C/C) at higher frequency and in heterozygosity (T/C) at lower frequency in sALS patients, as compared to controls (0.2 vs 0.06 and 0.1 vs 0.3, respectively). On the other hand, the c.1-100t>c polymorphism (rs968545; NG_008734.1:g.4901T>C; NM_007375.3:c.-234T>C) was present only in heterozygosity (T/C) at higher frequency (0.2 vs 0.1) than in controls^52^.

**Figure 6:**
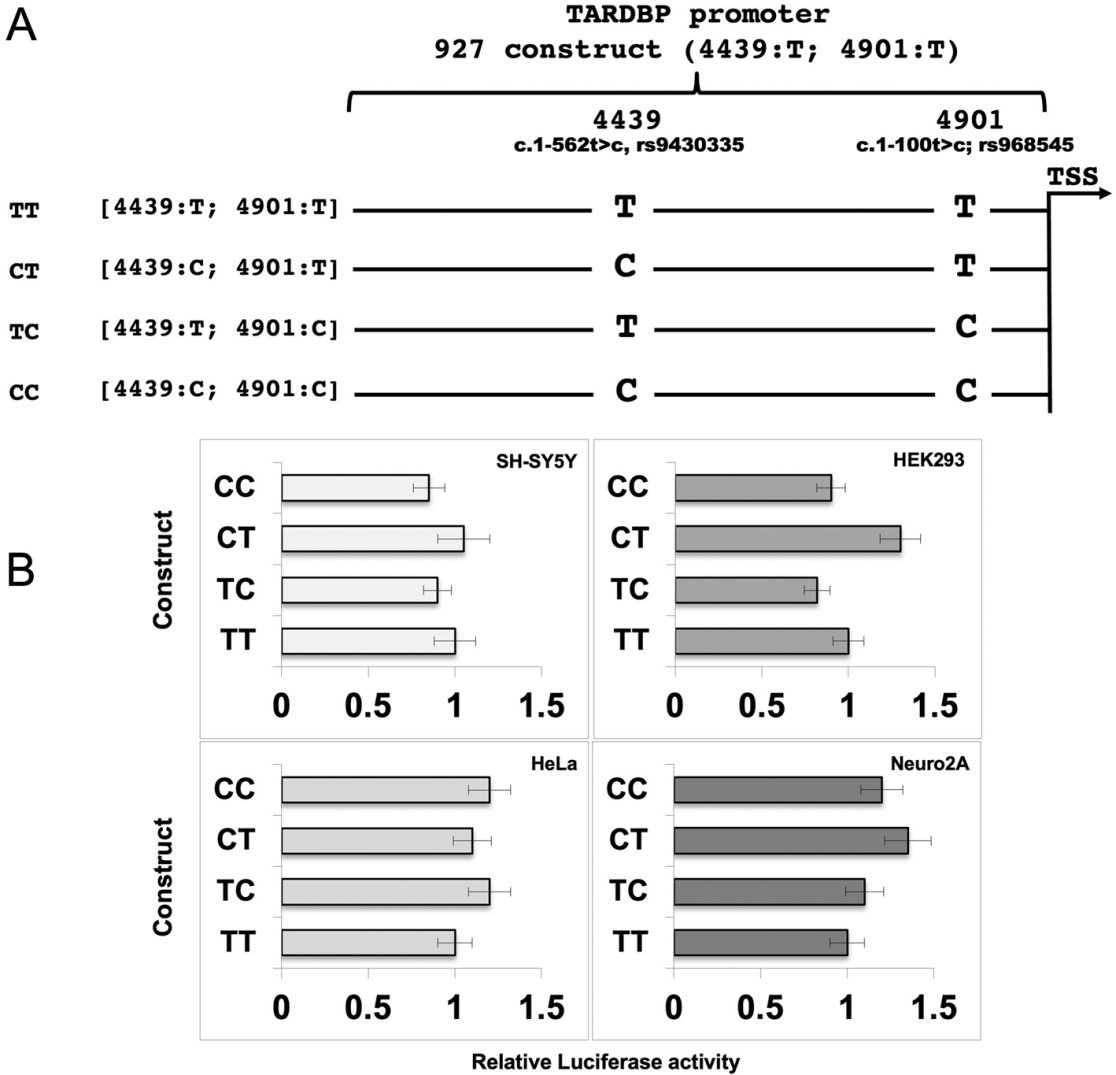
Effects of ALS-related SNPs on TARDBP promoter activity. (A) Schematic representation of the human TARDBP promoter variants. The diagram depicts the wild type and the mutated promoter sequences used for the luciferase assay. The mutated nucleotides (and their positions) are indicated. (B) Luciferase activity of TARDBP promoter variants in different cell lines (SH-SY5T, HEK293, HeLa and Neuro 2A). Luciferase activity (normalized against Renilla) is expressed as-fold against transfections of the haplotype [4439:T; 4901:T] (= 1). Data are mean (± SD) of three independent assays/cell line. The mean values did not differ to a statistically significant extent (p ≥ 0.05, one way ANOVA with Tukey test).

Therefore, we sought to test the impact of these SNPs on the transcription of TDP-43, in order to find a possible functional correlation with the manifestation of ALS. In order to analyze the SNP effects alone or in combination, we created three variants of our 927 original pGL4 construct (4439:T; 4901:T), so reproducing all the possible alleles (4439:T; 4901:C), (4439:C; 4901:C) and (4439:C; 4901:T) (Fig. 6A). The constructs were transfected in SH-SY5Y, HEK293, HeLa and Neuro2A cell lines. The luciferase activity of the three mutant constructs was compared to that of control (4439:T; 4901:T), used as unitary reference (Fig. 6B): no statistically significant differences were observed in the transcriptional activity of the three variants in all the tested cell lines (Fig. 6B).

### TDP-43 does not influence its own transcription

After characterizing the sequence of the promoter of TARDBP gene and its activity in different cell lines, we wished to evaluate the ability of TDP -43 to influence, directly or indirectly, the synthesis of its own transcript. For transfections of these constructs, a stable HEK293-flp-in cell line carrying a single copy of flag-tagged wild type human TDP-43 (HEK-TDP43wt) was used. This cell line was generated in our laboratory^44,60–62^ and was used as it allows a more homogenous TDP-43 expression from a Tetracycline inducible expression vector.

**Figure 7:**
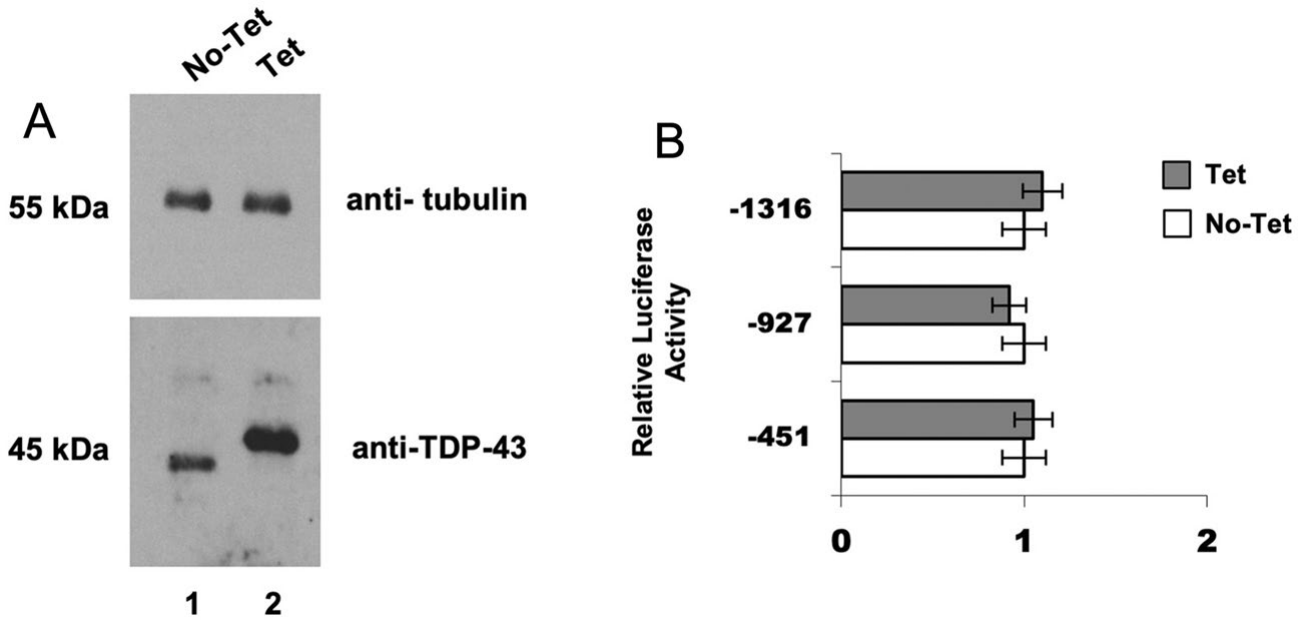
Effects of TDP-43 overexpression on TARDBP promoter activity. (**A**) Immunoblot analysis of TDP-43 expression after 48 h-Tetracycline induction (Tet) of the HEK293-Flp-In-TDP-43 wild type stable cell line. Both endogenous (lane 1) and Flag-tagged (lane 2) proteins were visualized using anti-TDP-43 polyclonal antibody (ProteinTech). As expected, overexpression of Flag-tagged wild-type TDP-43 silenced the expression of the endogenous protein by triggering autoregulatory loop. Beta-Tubulin was used as loading control to normalize the levels of detected proteins. (**B**) Effects of TDP-43 overexpression on TARDBP promoter activity. The luciferase activity of three different constructs (1316, 927 and 451) transfected in the HEK293-Flp-In-TDP-43 wild type stable cell line was assayed without (No-Tet) or after (Tet) Tetracycline induction for 48 h. No statistically significant differences were found. Data are mean (± SD) of three independent assays. Significance values refer to comparisons against control transfections (No-Tet) by using one way ANOVA with Tukey test.

The HEK-TDP43wt cells were transfected with the constructs 451, 927, and 1316 (along with pRL-TK plasmid). Subsequently, TDP-43 expression was driven by Tetracycline induction (48 h) and its levels were probed by Western blotting (Fig. 7A). Uncropped images for western blots shown in Fig. 7A are shown in Supplementary Fig. 1. As expected, Tetracycline induction of the Flag-tagged wild-type TDP-43 caused reduction of endogenous protein expression by triggering the known autoregulatory loop (Fig. 7A, lane 2). However, analysis of the luciferase activity 48 h later did not show significant differences in promoter activity upon TDP-43 tetracycline-induced overexpression (Fig. 7B). Similarly, no significant differences were observed after transient transfection of SH-SY5Y with the constructs 451, 927, and 1316 (with Renilla reporter) after TDP-43 transient overexpression (data not shown). These results suggest that TDP-43 does not influence its own transcription.

### The TARDBP 5′UTR and intron 1 splicing positively impact the luciferase expression

**Figure 8:**
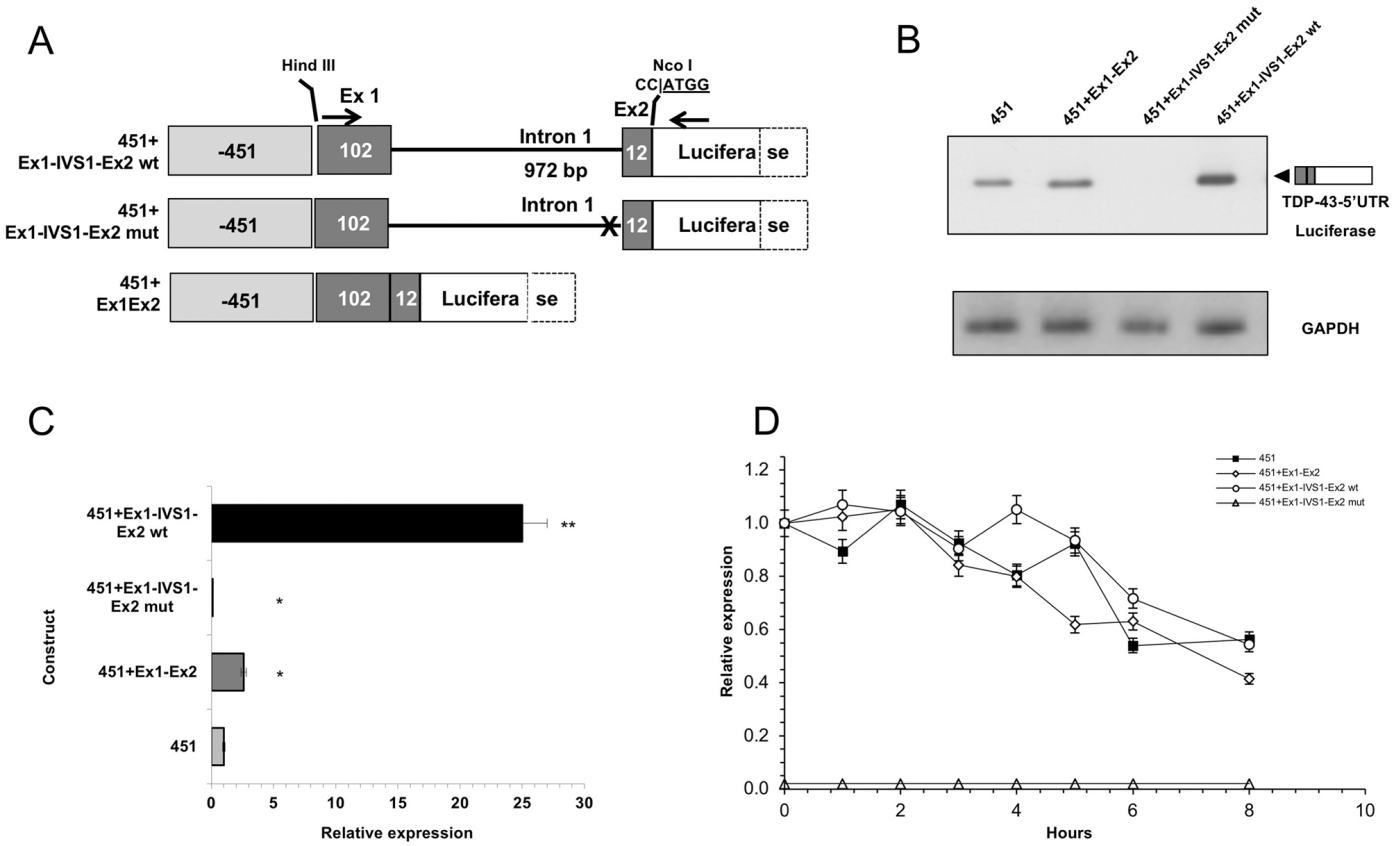
Effects of 5′UTR and intron 1 on the transcriptional activity of the human TARDBP promoter. (**A**) Diagrams of the 5′UTR TDP-43 constructs generated using the pGL-451 vector. In the 451 + Ex1-IVS1-Ex2 wt construct, the 1086 bp genomic region of the human TARDBP gene spanning the 5′UTR, including Exon 1 (102 bp), Intron 1 (972 bp) to the first 12 nt of Exon 2) was (Hind III-Nco I) cloned in between the 451nt-promoter and the firefly luciferase ORF (Luciferase). In the 451 + Ex1-IVS1-Ex2 mut construct, the 3′ splicing site of Intron 1 was deleted. In the 451 + Ex1Ex2 construct, the fragment encompassing Exon 1 and Exon 2 (without Intron 1) was cloned in the 451 vector. (**B**) RT-PCR of the 5′UTR TDP-43 mRNA species (upper panel). Amplification of GAPDH (lower panel) was used as the endogenous control in the quantitative analysis of RT-PCR. (**C**) Quantitation of the 5′UTR TDP-43 mRNA species by Real Time PCR. GAPDH was used to confirm normalization of total RNA levels. Co-transfected renilla orf was used to normalize the luciferase qPCR. The 451 construct was used as reference (= 1). (**D**) Effects of transcriptional inhibition on mRNA stability. After 36 h post-transfection of the luciferase (451 + Ex1-IVS1-Ex2 wt and 451 + Ex1-IVS1-Ex2 mut) constructs, SH-SY5Y cells were treated with actinomycin D 5 µg/ml. The relative levels of the indicated mRNAs (wt and mut) were assessed at the designated time points, following shutoff of transcription using qRT-PCR to determine mRNA half-lives. The average half-lives are reported with SD from two independent experiments. Significance values refer to comparisons against t = 0 h, wt: *p < 0.05 (one way ANOVA with Tukey test). The mean values for 451 + Ex1-IVS1-Ex2 mut construct were negligible and did not differ to a statistically significant extent (p ≥ 0.05).

The 5′UTR region of TARDBP gene encompasses exon 1 (102 bp) and the first 12 bp of exon 2, separated by intron 1 (972 bp). In order to explore the presence of additional elements able to modulate TDP-43 expression, the functional impact of the 5′ UTR (construct 451 + Ex1Ex2) and intron 1 (construct 451 + Ex1-IVS1-Ex2) of *TARDBP* was analysed by generating variants of the 451 plasmid (Fig. 8A). A first variant was containing the TARDBP 5′UTR (exon 1, 102 bp and exon 2, 12 bp) correctly spliced (451 + Ex1Ex2). The second construct was created by inserting the region encompassing exon1 (102 bp), intron 1 (972 bp) and the first 12 bp of exon 2 of TARDBP gene in between the 451 promoter and the luciferase ATG codon (451 + Ex1-IVS1-Ex2 wt). The third construct was a mutant of latter construct where the 3′ splice site of intron 1 was disrupted (451 + Ex1-IVS1-Ex2 mut).

**Figure 9:**
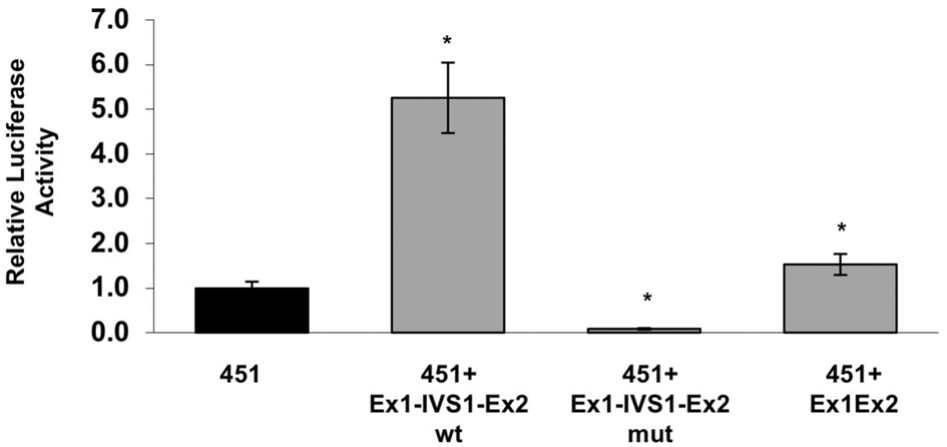
Effects of 5′UTR and intron 1 on the luciferase activity of the TARDBP promoter constructs. The luciferase activity of 5′UTR TDP-43 constructs depicted in Fig. 8A was assayed by transient cotransfections (luciferase and renilla) in SH-SY5Y cells. After 48 h, cells were assayed for firefly luciferase expression (normalized against renilla). The empty expression vector was tested and produced background levels of luciferase activity (data not shown). Data are mean (± SD) of three independent assays. Significance values refer to comparisons against control transfections (451): *p < 0.05 (one way ANOVA with Tukey test).

In SH-SY5Y cells, the presence of a correctly sized amplicon was observed only after transfection of the 451, 451 + Ex1Ex2 and 451 Ex1-IVS1-Ex2 wt constructs (Fig. 8B). Uncropped images for agarose gel shown in Fig. 8B are shown in Supplementary Fig. 2. Quantitation of the mRNA species was determined by Real Time PCR (Fig. 8C). By using the 451 construct as the reference (= 1), the relative expression of the 451 + Ex1Ex2 (containing the pre-arranged and correctly spliced 5′UTR region) was 2.6 × that of the reference, while the 451 + Ex1-IVS1-Ex2 wt construct showed a 25 × increment in relative expression compared to the -451 construct). Conversely, the mRNA levels of the 451 + Ex1-IVS1-Ex2 mut construct were negligible (0.1 × vs 451 control). In order to test if mutant transcript is unstable due to low efficiency of splicing, the RNA level of 451 + Ex1-IVS1-Ex2 wt and 451 + Ex1-IVS1-Ex2 mut constructs were monitored with and without addition of a transcriptional inhibitor (Actinomycin D) that causes complete transcriptional arrest^63^. Considering the higher mRNA levels derived from the intron-containing construct (451 + Ex1-IVS1-Ex2 wt), to prevent the possibility that the decay machinery might be saturated by the spliced mRNAs, we used proportionately less of this construct, while keeping constant the amount of total transfected DNA. While it was not possible to determine the half-life of the 451 + Ex1-IVS1-Ex2 mut mRNA (whose expression was negligible), the mRNA species of both the 451 + Ex1-IVS1-Ex2 wt and the 451 + Ex1Ex2 constructs exhibited half-lives of ∼ 7 h, indicating no significant differences in mRNA stability dependent on splicing (Fig. 8D). Lastly, the relative luciferase activities positively correlate with the occurrence of splicing. In fact, the same constructs were transfected in SH-SY5Y cells and their luciferase activity was measured (Fig. 9): the activity of the 451 + Ex1Ex2 wt (containing the pre-arranged and correctly spliced 5′UTR region) was 1.5 × that of the control. The 451 + Ex1-IVS1-Ex2 wt construct showed a 5 × increment in luciferase activity (as compared to the control, -451 construct). On the other hand, as expected, the 451 + Ex1-IVS1-Ex2 mut construct showed insignificant activity when compared with the other constructs (Fig. 9). Altogether these results suggest that the presence of the 5′UTR as well as the correct splicing event of intron 1 are elements able to modulate the luciferase expression (and, potentially, of TDP-43) at transcriptional level.

## Discussion

We have experimentally characterized the promoter region of the human TARDBP gene. A previous prediction indicated that the core regions of the TARDBP promoter could be located between 500 nucleotides upstream of exon 1 and 66 nucleotides downstream of exon 1 and in two regions of intron 1 (212 and 613 nucleotides)^52^. Our predictions failed to identify the canonical TATA box and CAAT box transcriptional regulatory elements and interrogation of the database DBTSS (cataloging the positions of TSSs in the genome) outlined how, in most TDP-43 transcripts, transcription starts from the TSS indicated in the main transcript isoform NM_007375. Regarding the spatio-temporal expression pattern, TDP-43 is a developmentally regulated protein^58,64^, while its distribution in adult tissues seems to be ubiquitous^6,58,64^. These observations are consistent with the hypotheses supported by recent studies reporting that TATA box is present in only a minority of promoters^65–67^, often with tissue-specificity such as liver and muscle^68–70^.

From an evolutionary perspective, it is interesting to note that, among primates, the greatest degree of evolutionary conservation encompasses the 600nts upstream of the TSS. On the other hand, the alignment of human and rodents sequences highlights the 200nts upstream of the TSS as the region of higher homology. These observations suggested that the region in-between plays an important role in directing transcription of the TARDBP gene. Indeed, this hypothesis is consistent with the experimental mapping performed in this study that has outlined the region spanning nucleotides 451–230 as necessary for the minimal promoter activity in all the tested cell lines (Fig. 4).

The comparison of the transcriptional activity in different cell lines highlights how the TARDBP promoter activity is high in cell lines of neuronal origin as well as in HeLa, as compared to the human embryonic kidney 293 cells (Fig. 5). These observations show that the promoter is active in human and mouse cell lines of different tissue origin (as expected) and suggest that apparent relative lower activity observed in HEK293 cells might rely on biochemical and/or genomic factors associated to this specific cell line. In addition, it should be also considered that, in comparison with other tested cell lines, HEK293 cell line has no evident tissue-specific gene expression and show expression of markers of several tissues^71^.

It is well established that neuronal TDP-43 cytoplasmic inclusions are present in several different neurodegenerative diseases, such as FTD, ALS and Alzheimer and that the sequestration of this protein in aggregates may create gain- and loss-of-function events causing (directly or indirectly) cellular toxicity or alteration of the TDP-43-regulated gene expression. Understanding the factors that control TDP-43 expression within cells will certainly provide a better insight into disease origin and progression. Indeed, it has been observed that an increase of TDP-43 protein levels can activate the aggregation process in both cytoplasmic and nuclear compartments^20,60^. In addition, studies with animal models have shown that TDP-43 overexpression can be neurotoxic, even without the presence of aggregates^30,32,33,72,73^. Indeed, although TDP-43 can regulate its own expression through a negative feedback loop^44^, some observations suggest that regulation of its expression might may also occur at transcriptional level. In particular, TDP-43 expression seems to be developmentally regulated^64^, but also TDP-43 expression has been shown to decrease with age in a variety of organisms such as fruit fly^59^ and mouse^58^.

Looking for potential modifiers of the TARDBP promoter activity, we have focused the attention on two SNPs (c.1-562t>c, rs9430335 and c.1-100t>c, rs968545) previously identified within the TARDBP promoter of SALS patients. Although the variants do not change any transcription factor binding site, we have functionally tested their transcriptional impact. Although, in our system, we did not find statistically significant differences in the promoter activity of each haplotype, we can hypothesize that the potential regulatory SNPs do not lead to variation of TARDBP promoter activity directly, but cannot exclude that other possibilities of how these may affect transcription levels do exist. The variants might affect transcription at endogenous copy numbers, but not for overexpressed exogenous constructs. In addition regulatory elements, outside the promoter region included in our reporter plasmid and might interplay with the variants and modulate the activity of the promoter.

Subsequently, since TDP-43 autoregulates its own expression through a negative feedback mechanism via direct interaction with its own 3′UTR^44,48,74,75^, we verified if there are transcriptional effects of TDP-43 on its own promoter activity and it does not modulate its own transcription.

Then, considering the known ability of the 5′UTRs to affect mainly protein translation efficiency^76^, we next sought to test the possible effects of the TARDBP 5′UTR and intron 1 splicing on the expression of the associated reporter gene (Fig. 8).

On one hand, the observation of a significant increase in luciferase activity associated with the presence of the TARDBP "pre-arranged" 5′UTR (construct 451 + Ex1Ex2) indicated that this element can positively influence gene expression. Consistently, recent studies have shown that approximately 35% of human 5′UTRs are annotated as harboring introns^77^ and recent observations suggest that introns in UTRs may have specific regulatory functions by affecting the rate of transcription rather than transcript stability. On the other hand, our experiments aimed at testing the relative mRNA decay rates of the luciferase constructs indicate that these elements have little or no impact on the stability of the mature mRNA, and therefore, they might affect gene expression through modulation of other steps of RNA metabolisms, such as transcription, mRNA levels, mRNA export, translational efficiency^78–81^.

In conclusion, growing lines of evidence suggest that the pathophysiology of ALS, FTD and other neurodegenerative diseases might be the consequence of the convergence of multiple risk factors acting also on changes of TDP-43 levels. Indeed, TDP-43 expression can be modulated during development^64^, in a cell specific fashion, and, through aging, these fluctuations are evolutionary conserved at least in mice and flies^58,59^. Notwithstanding there are no clear data of age-related changes in TDP-43 in human^82,83^, altogether these observations suggest that a better understanding of the mechanisms and factors involved in controlling the activity of the TARDBP promoter and, consequently, TDP-43 expression will give clues to uncover novel processes implicated in the onset and progression of the TDP-43 proteinopathies.

## Methods

### Constructs

The 1316 bp region upstream of the TARDBP TSS was amplified by PCR and cloned in pGEM-T easy vector. The 1316 bp insert was digested with the restriction enzymes KpnI and HindIII (these sites were designed into the primers used in PCR) and subcloned into the pGL4.1 vector (Promega, Madison, WI, USA) digested with the same restriction enzymes. Other 7 deletion-mutants of TDP-43 promoter (927, 451, 380, 330, 280, 230, 180 bp upstream of the TSS) were then generated by PCR using gene-specific forward primers and the pGL4-1316 construct as template. Deletion-mutant inserts were KpnI-HindIII-subcloned in the pGL4.1 vector. TARDBP promoter SNPs were generated by PCR mutagenesis. The identity of all constructs was verified by DNA sequencing. Primer sequences are available.

### Transfections and luciferase assays

The cell lines used for transfections were the following: HEK293, HeLa, Neuro2A, and SH-SY5Y. Each construct was transfected in duplicate, 600 ng of DNA was diluted in a final volume of 100 µl with Opti-MEM I Reduced Serum Medium (ThermoFisher, Waltham, MA, USA). To normalize for transfection efficiency, the cells were co-transfected with 20 ng/well of pRL-TK (Promega, Madison, WI, USA) plasmid along with the promoter constructs. In parallel, 98 µl of Opti-MEM were added to 2 µl Lipofectamine 2000 Reagent (ThermoFisher, Waltham, MA, USA). After 5 min. at room temperature, the two solutions were combined and mixed, and 20 min later, the growth medium was removed from the cells and replaced with fresh DMEM containing 5% FBS. Then, the transfection mix was added to each well.

Total cell lysate was prepared from cells 48 h post transfection and firefly luciferase activity was assayed using the Beetle-Juice KIT (P.J.K. GmbH, Kleinblittersdorf, Germany) and a Turner Design 20/20 Luminometer (Turner BioSystems, Sunnyvale, CA, USA). Control transfections with pGL4.1 empty vectors were used as negative controls. Data presented are mean ± Standard deviation (SD) of at least three independent experiments and given as fold expression overexpression indicated in the Figures set arbitrarily at 1. Statistical analysis was performed using Student’s T or ANOVA tests. In order to verify the possible influence of TDP-43 on its own promoter, we used the previously generated human stable cell line Flp-In HEK293 -TDP-43 wild type, carrying the human TDP-43 transgene locus-specific-integrated, whose expression is driven by an inducible version of the Tetracycline (Tet) promoter^44^.

One day before transfection, Flp-In HEK293 -TDP-43 wild type (wt) cells were seeded at 60% confluence. Lipofectamine reagent was used for transfection with 300 ng of the reporter plasmids and 20 ng of pRL-TK vector. After 12 h, the medium was changed, and the cells were incubated further for 24 h. Flag-tagged TDP-43 expression was induced with 1 µg/ml tetracycline. The luciferase/renilla activities were then assayed and statistical analyses were performed using Anova one-way followed by Tukey's test.

### Gene expression quantitation

In order to quantify the mRNA levels of the 5′UTR TDP-43 constructs, co-transfections of luciferase constructs and pRL-TK were carried out as described in the section "Transfections and Luciferase assays".

Relative expression of constructs 451, 451 + Ex1Ex2, 451 Ex1-IVS1-Ex2 wt and 451 Ex1-IVS1-Ex2 mut constructs was performed by semi-quantitative RT–PCR and by quantitate Real Time PCR. Forty eight hours after transfection, cultures were terminated and RNA was extracted with Trifast reagent (Euroclone, Milan, Italy), according to manufacturer’s instruction. In the semi-quantitative RT–PCRs, GAPDH was used as internal control. The sequences of primers were (5′–3′): GAPDH_Ex1s, 5′-CGCTCTCTGCTCCTCCTGTT-3′; GAPDH_Ex2as, 5′-CCATGGTGTCTGAGCGATGT-3′; TDP-43 Ex1 + 9 (277 bp)_s, 5′-AGCTTGCGCCATTTTGTGGGAGCGA-3′; Luc ATG + 155(277 bp)_as, 5′-GTAATGTCCACCTCGATATGTGCGT-3′. Optical density of amplicons was calculated using the ImageJ image processing program^84^ and the expression levels were numerically expressed as the ratio of applicant density of the target amplicons over that of GAPDH.

Quantitation of the 5′UTR TDP-43 mRNAs by Real Time PCR, co-transfections of luciferase constructs and pRL-TK were carried out as described in the section "Transfections and Luciferase assays". The extracted RNA (1 µg) was DNAse-treated and reverse-transcribed using the M-MLV reverse transcriptase (Thermo Fisher Scientific, Waltham, MA, USA), and oligo-dT (0.2 µg/reaction as primer), in a final volume of 40 µl, essentially as indicated in the manufacturer's instructions. The CFX96 real-time PCR detection system (Bio-Rad Laboratories, Redmond, WA, USA) was used for all qPCRs. SYBR green assays were optimized (annealing temperature, and incubation time) with the iQ SYBR Green Supermix (Bio-Rad), according to the manufacturer's instructions, using tenfold serial dilutions of cDNAs. The amplification mixture consisted of 0.2 μM primers, 5 μl of SYBR Green Supermix, and 4 μl template DNA in a total volume of 10 μl. Samples were amplified with the following program: initial denaturation at 95 °C for 2 min, followed by 40 cycles of denaturation for 15 s at 95 °C and annealing/elongation for 30 s at 60 °C. The expression of the co-transfected renilla orf was used to normalize the luciferase qPCR data.

Primer sequences were: Luciferase (62 bp), forward 5′-CACAAAGCCATGAAGCG-3′ and reverse 5′-ATATGTGCGTCGGTAAAG-3′; Renilla (140 bp), forward 5′-CAGAACAAAGGAAACGGATGA-3′ and reverse 5′-CGCGTTACCATGTAAAAAA-3′.

Melting curves were examined to determine the specificity of each product and relative expression data were calculated with the ΔΔCT method^85^.

For experiments with actinomycin D, SH-SY5Y cells were transfected with 3000 ng of no-intron constructs (451, 451 + Ex1Ex2) or of the 451 Ex1-IVS1-Ex2 mut plasmid or 600 ng of the 451 Ex1-IVS1-Ex2 mut construct in a p100 plate. Total DNA was normalized to 3000 ng by adding an appropriate amount of pUC18 vector. Twelve hours later, cells deriving from each transfection were harvested and plated in different wells (one for each actinomycin incubation time point). Twenty-four hour after plating, actinomycin D (5 μg/ml) was added. The relative levels of the indicated mRNAs were assessed at the designated time points, following shutoff of transcription using qRT-PCR. Samples were collected at the indicated times using TRIzol. Total RNA from these samples was analyzed via qRT-PCR using the abovementioned Luciferase primers and normalization was carried out versus the housekeeping gene RPII (RPII_f: 5′-GCCCACGTCCAATGACAT-3′; RPII_r: 5′-GTGCGGCTGCTTCCATAA-3′). Relative transcript abundances were determined using the ΔΔCt method^85^.

### Western blot

Cells extracts were prepared in PBS containing 1 × protease inhibitor (Roche Diagnostics, Indianapolis, IN, USA). Proteins were separated by SDS-PAGE and transferred to nitrocellulose (Cytiva, formerly GE Healthcare, Chicago, IL, USA) and protein detection was carried out with standard Western blotting techniques. After transfer, membranes were incubated for 10 h in blocking solution (5% nonfat dry milk in PBS containing 0.1% Tween-20, T-PBS) to prevent non-specific binding. Subsequently, membranes were incubated for 1 h at room temperature with specific primary antibodies diluted in blocking solution. Expression levels of both endogenous and flag-tagged human wild-type TDP-43 was monitored by using a commercially available polyclonal anti-TDP-43 antibody (Proteintech, Rosemont, IL, USA, 10782-2-AP). Endogenous tubulin was used as a loading control, using an in-house made mouse monoclonal antibody. Immunoblots were developed by using the ECL Star Enhanced Chemiluminescent Substrate (EuroClone, Pero, Milan, Italy).

## Supplementary Information

Supplementary Figures.
