# Supplementary material for: Characterization of the human TARDBP gene promoter
Source: Sci Rep. 2021 May 17;11:10438. doi: 10.1038/s41598-021-89973-z (PMC8129075; doi:10.1038/s41598-021-89973-z)

Supplementary Figure 1

Uncropped images for western blots shown in Figure 7A

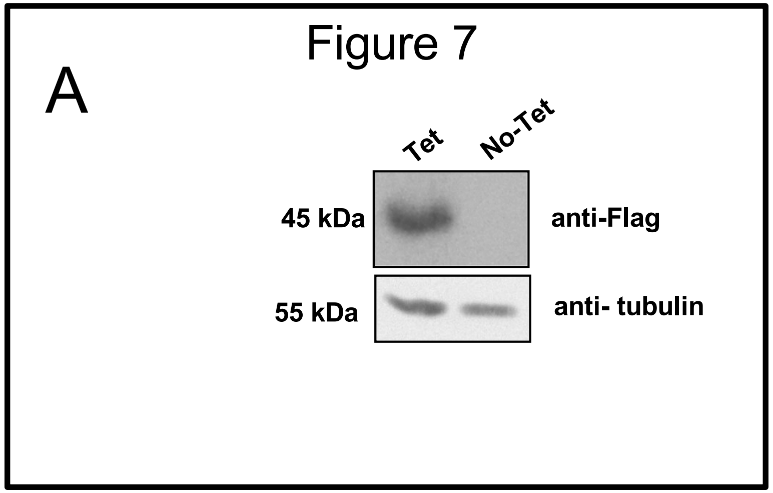

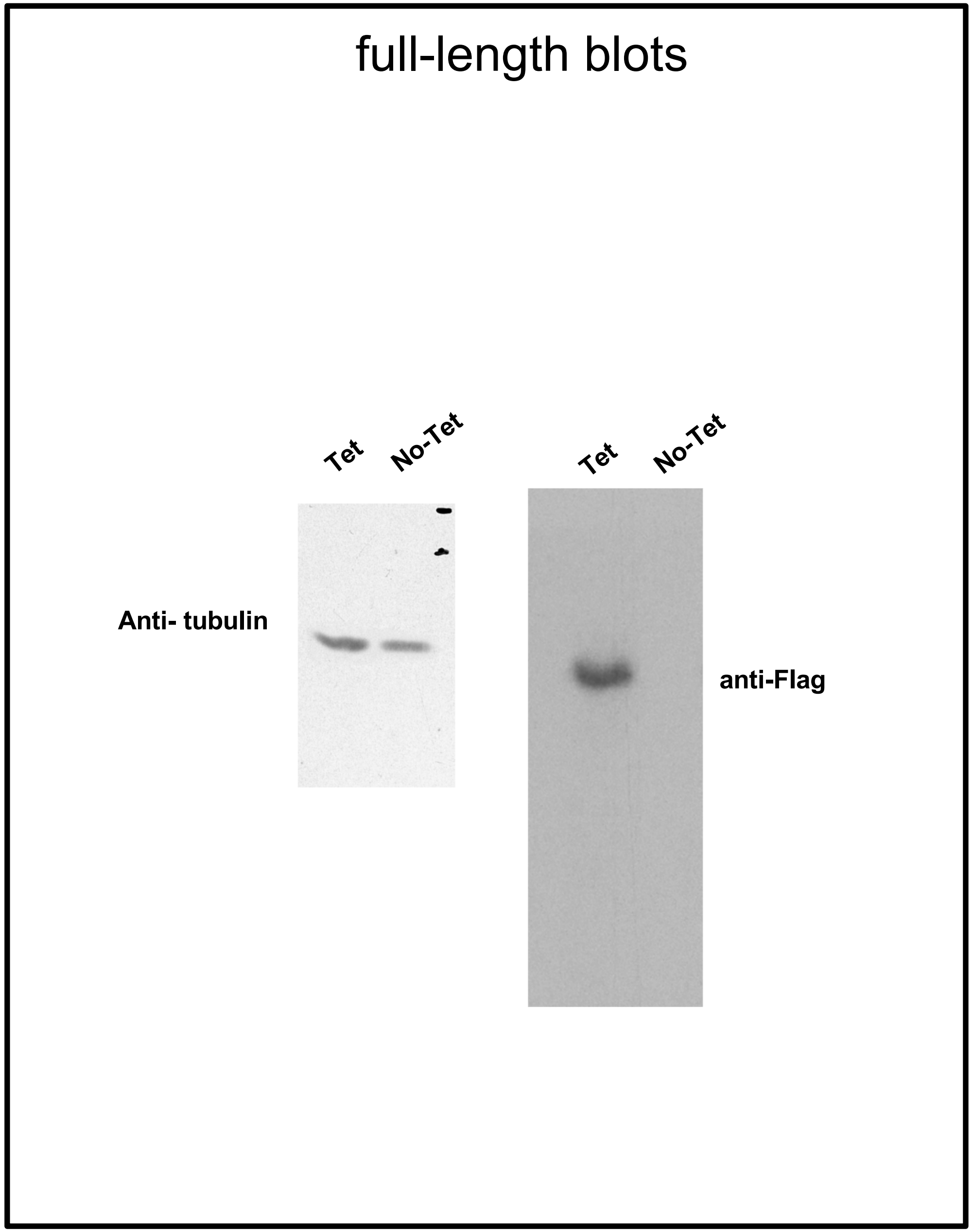

Supplementary Figure 2

Uncropped images for agarose gel shown in Figure 8B

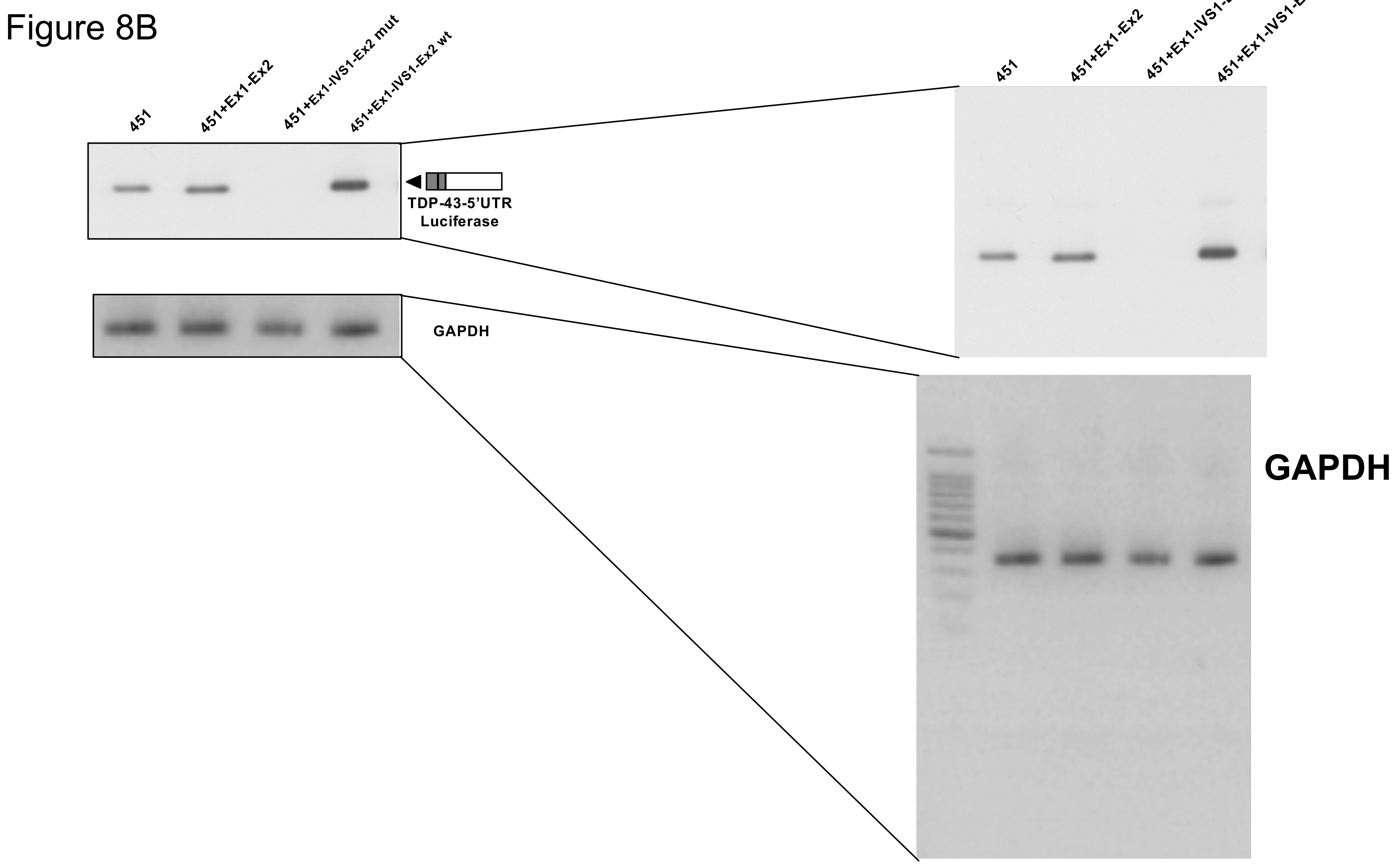

Supplement: Supplementary file 1 — Supplementary Figures. [file 41598_2021_89973_MOESM1_ESM.pdf]
